# Poly(vinyl alcohol)-tannic Acid Cryogel Matrix as Antioxidant and Antibacterial Material

**DOI:** 10.3390/polym14010070

**Published:** 2021-12-25

**Authors:** Betul Ari, Mehtap Sahiner, Sahin Demirci, Nurettin Sahiner

**Affiliations:** 1 Department of Chemistry, Faculty of Science & Arts, Terzioglu Campus, Canakkale Onsekiz Mart University, Canakkale 17100, Turkey; betullcan@gmail.com (B.A.); sahindemirci@gmail.com (S.D.); 2 Faculty of Canakkale School of Applied Science, Terzioglu Campus, Canakkale Onsekiz Mart University, Canakkale 17100, Turkey; msahiner@comu.edu.tr; 3 Nanoscience and Technology Research and Application Center, Terzioglu Campus, Canakkale Onsekiz Mart University, Canakkale 17100, Turkey; 4 Department of Chemical and Biomolecular Engineering, University of South Florida, Tampa, FL 33620, USA; 5 Department of Ophthalmology, Morsani College of Medicine, University of South Florida, 12901 Bruce B Downs B. Downs Blv., MDC 21, Tampa, FL 33612, USA

**Keywords:** cryogel, antioxidant, antibacterial, polyvinyl alcohol, phenolic compound

## Abstract

The biocompatible, viscoelastic properties of poly(vinyl alcohol) (PVA) in combination with the antimicrobial and antioxidant natural polyphenolic, tannic acid (TA), and the natural flavonoid and antioxidant curcumin (Cur), were used in the preparation of PVA:TA and PVA:TA:Cur cryogel composites using cryotropic gelation to combine the individually beneficial properties. The effect of TA content on the antioxidant and antimicrobial activities of PVA:TA cryogel composites and the antioxidant activities of PVA:TA:Cur cryogel composites was determined using Trolox equivalent antioxidant capacity (TEAC) and total phenol content (TPC) assays, and were compared. The PVA:TA:Cur cryogel composite showed the highest antioxidant activity, with a TEAC value of 2.10 ± 0.24 and a TPC value of 293 ± 12.00. The antibacterial capacity of the PVA:TA and PVA:TA:Cur 1:1:0.1 cryogel composites was examined against two different species of bacteria, *E. coli* and *S. aureus*. It was found that the minimum inhibition concentration (MIC) value of the PVA:TA:Cur 1:1:0.1 cryogel composites varied between 5 and 10 mg/mL based on the type of microorganism, and the minimum bactericidal concentration (MBC) value was 20 mg/mL irrespective of the type of microorganism. Furthermore, the hemocompatibility of the PVA:TA cryogel composites was evaluated by examining their hemolytic and coagulation behaviors. PVA:TA 1:1 cryogels with a value of 95.7% revealed the highest blood clotting index value amongst all of the synthesized cryogels, signifying the potential for blood contacting applications. The release of TA and Cur from the cryogel composites was quantified at different pH conditions, i.e., 1.0, 7.4, and 9.0, and additionally in ethanol (EtOH) and an ethanol–water (EtOH:Wat) mixture. The solution released from the PVA:TA cryogels in PBS was tested for inhibition capability against α-glucosidase (E.C. 3.2.1.20). Concentration-dependent enzyme inhibition was observed, and 70 µL of 83 µg/mL PVA:TA (1:1) cryogel in PBS inhibited α-glucosidase enzyme solution of 0.03 unit/mL in 70 µL by 81.75 ± 0.96%.

## 1. Introduction

Poly(vinyl alcohol) (PVA) is a synthetic non-toxic polymer that has been used primarily for textile and packaging applications owing to its ductile mechanical properties and efficient film formation ability [1,2,3]. Recently, the use of PVA-based materials in pharmaceutical and biomedical fields has gained accelerated interest, and they are proposed as promising candidates for the entrapment of cells, delivery applications [4], wound healing materials [5,6], as well as for the replacement of cartilage and cardiac valves [7,8].

Tannic acid (TA) is a natural polyphenol, is inexpensive, water soluble, and has low-toxicity [9,10], with numerous bioactive and health-promoting effects such as antioxidant, antimicrobial, and anti-inflammatory properties, enabling many diverse biological uses [11,12]. Because of these properties, TA has been the focus of many studies for the production of many biochemical products, pharmaceuticals, membrane technologies, cosmetics, and for biomedical applications [13,14,15]. Curcumin (Cur), also known as turmeric, is also a polyphenol obtained from the underground roots of the plant, namely, rhizomes [16,17]. Additionally, Cur is a hydrophobic natural pigment with many well-known clinical and biological applications due to its beneficial and broad range of pharmacological and biological characteristics, e.g., antibacterial, antioxidant, antimalarial, anti-inflammatory, and anti-HIV properties. Cur is proven to be non-toxic and is a potential anti-carcinogenic, and is a pharmacological material with great medical application potential because of these bio-beneficial properties [18,19,20,21,22,23,24]. However, several disadvantages, such as low bioavailability, low solubility in water, lipophilic structure, and fast metabolism rate limit its clinical applications [25,26,27]. Therefore, the synthesis method chosen here is cryogelation in the preparation of cryogel composites, as it is easy to apply, simple, and adaptable for the incorporation of delicate materials such as Cur as it is done below the freezing points of the used solvents (<0 °C for water).

Cryogels are super macroporous three-dimensional polymer networks with superior physical properties compared to conventional hydrogels made of the same materials. The most important features that distinguish cryogels from hydrogel equivalents are that they are highly porous materials with better mechanical strength and faster response times to external stimuli. It is possible to synthesize cryogel with other materials in composite formulations to further improve the innate properties [28,29,30,31,32,33,34,35]. For this purpose, PVA, a non-toxic biocompatible synthetic polymer with excellent physico-mechanical and viscoelastic properties [1,2,3,36,37], and a natural polyphenol TA and Cur possessing antioxidant, anti-inflammatory, anticancer, and antimicrobial activities [11,22] were used to design PVA:TA and PVA:TA:Cur cryogel composites. PVA:TA cryogel composites, differing in the quantity of TA content, with 1:0.1, 1:0.25, 1:0.5, and 1:1 by weight of PVA, and PVA:TA:Cur cryogel/composite with Cur content of 1:0.1 by weight of PVA, were prepared using the cryotropic gelation method. Antioxidant and antimicrobial properties imparted by the incorporation of TA and Cur moieties were determined, and the interactions of PVA:TA and PVA:TA:Cur cryogel composites with biological interfaces, e.g., blood, were examined by evaluating their hemolytic and coagulation behaviors. Furthermore, the kinetics of TA release from the PVA:TA cryogel composites in media with varying pH were investigated by conducting in vitro release studies, e.g., at pH 1.0, pH 7.4, and pH 9.0. Cur and TA release profiles from PVA:TA:Cur 1:1:0.1 cryogel composites were also tested in ethyl alcohol (EtOH) and EtOH–water mixture.

## 2. Materials and Methods

### 2.1. Materials

Poly(vinyl alcohol) (PVA, MW = 89–98 kg/mol, ≥99%, Sigma-Aldrich, St. Louis, MI, USA), tannic acid (TA, purity meets analytical specification of USP, Sigma-Aldrich, Overijse, Belgium), curcumin (Cur, from *Curcuma longa* (*Turmeric*)) powder, Sigma-Aldrich, Shanghai, China), and sodium hydroxide (NaOH, Merck, Darmstadt, Germany) were used as received without further purification. Ethyl alcohol (EtOH, ≥99.9%, Carlo Erba, France) was used as supplied. In the antioxidant activity determination studies, Folin Ciocalteau phenol reagent (FC, 2N, Sigma-Aldrich, Schaffhausen, Switzerland) and sodium acetate (Na_2_CO_3_, 99%, Fisher Scientific, Schwerte, Germany) were used for the TPC test and 2,2′-Azino-bis(3-ethylbenzothiazoline-6 -sulfonic acid) diammonium salt (ABTS^.+^, 98%, Sigma-Aldrich, Oakville, ON, Canada) and potassium persulfate (KPS, 99%, Carlo Erba, Val-de-Reuil, France) were used for the TEAC test. Strains of bacteria, *E. coli* ATCC 8739 and *S. aureus* ATCC 25323, were procured from the Microbiology Department of the School of Medicine at Canakkale Onsekiz Mart University. For the enzyme studies, α-glucosidase from *Saccharomyces cerevisiae* (100 units/mg, Sigma-Aldrich, Israel), p-nitrophenyl-α-D-glucopyranose (p-NPG, 99%, Acros, Stabio, Switzerland), and potassium phosphate monobasic (98–100.5%, Sigma-Aldrich, Darmstadt, Germany) were purchased. All aqueous solutions were prepared using double-distilled water (DI, GFL 2108 water still, Berlin, Germany).

### 2.2. Synthesis of PVA Cryogel, PVA:TA, and PVA:TA:Cur Cryogel Composites

The synthesis of PVA cryogel and cryogel composites was carried out by making several modifications to the method reported in the literature [38]. The synthesis of PVA cryogels was achieved using 5 mL 0.176 g/mL PVA solution by dissolving PVA in 0.2 M NaOH at 120 °C for 15 min. Then, the PVA solution was allowed to cool down to 90 °C, quickly pipetted into plastic straws with a diameter of 8 mm, and kept in cryogenic conditions in a freezer at −18 °C for the cryogelation process for 24 h.

For the preparation of PVA:TA cryogel composites, TA in variable weight with respect to PVA, e.g., at 1:0.1, 1:0.25, 1:0.5, and 1:1 by weight of PVA, was dissolved in 0.2 M 5 mL NaOH solution. Next, 0.88 g of PVA in powder form was added into each solution and vortexed at a 1000 rpm stirring speed for 5 s. From here on, the same cryogelation steps were followed as stated above in the preparation of the PVA cryogels. After the period of cryogelation was complete, the cryogel composites were allowed to thaw at room temperature until all of the ice crystals melted, after which the cryogel composites were placed back in the freezer at −18 °C. The freeze–thaw process was repeated once a day for 7 days and the samples were washed once with water, freeze-dried, and stored until further use.

For the preparation of PVA:TA:Cur at 1:1:0.1 wt ratios of cryogel composites, Cur corresponding to 1:0.1 by weight of PVA was weighed and dissolved in a light-proof bottle in 0.2 M 5 mL NaOH solution. Then, TA corresponding to 1:1 by weight of PVA was weighed and added to the Cur solution and mixed to dissolve the TA. Finally, 0.88 g PVA was added to the TA:Cur solution and kept at 120 °C for 15 min to dissolve the PVA. Then, the temperature was allowed to cool down to 90 °C. The cooled cryogel/composite solutions were vortexed and immediately transferred to 8 mm diameter plastic straws and kept in the freezer at −18 °C for 24 h for the cryogelation process.

### 2.3. Characterization of PVA:TA and PVA:TA:Cur Cryogel Composites

The functional groups on all PVA:TA and PVA:TA:Cur cryogel composites were determined with a Fourier transform infrared radiation spectroscopy analysis (FT-IR, Perkin Elmer Spectrum100, Perkin Elmer Instruments, Norwalk, CT, USA) using the ATR technique with 4 cm^−1^ discrimination power in the range of 4000–650 cm^−1^.

The thermal stabilities of the synthesized cryogel composites against temperature were compared with a Thermogravimetric Analyzer (TGA, SII TG/DTA 6300, Seiko Ins. Corp, Chiba, Japan) device. About 3 mg of dried cryogel/composite pieces were placed in TGA cuvettes and heated from 25 °C to 100 °C under N_2_ gas atmosphere to remove moisture. Later, the temperatures of the dehumidified cryogels were increased from 75 °C to 1000° at 10 °C/min and 200 mL/min of N_2_ gas flow rate, and the weight loss% against temperature was recorded.

The morphological structures of the synthesized cryogels were determined using scanning electron microscopy (SEM, QUANTA 400F Field Emission, Hillsboro, OR, USA). To obtain SEM images, freeze-dried cryogels were finely cut and samples were adhered to carbon tapes. The samples were coated with Au-Pd under low vacuum and 2.0 kV voltage, placed on SEM stands, and SEM images were obtained.

The swelling behavior of the PVA cryogel, PVA:TA, and PVA:TA:Cur 1:1:0.1 cryogel composites in different pH conditions was investigated gravimetrically. Briefly, approximately 30 mg of dry PVA cryogel, PVA:TA, and PVA:TA:Cur 1:1:0.1 cryogel composite pieces were weighed. Then, these cryogel/composite pieces were swollen in a 10 mL volume of pH 7.4 buffer solution for 24 h. The swollen samples were removed from the buffer solution and lightly dried with filter paper to get rid of the excess surface water and were reweighed. The swelling ratio% (S%) and moisture capacity% (M%) of the cryogels were calculated using Equations (1) and (2), respectively. The same swollen cryogels were reweighed after gently squeezing between two fingers to determine the porosity of the cryogels. The porosity% (P%) of the cryogel samples was calculated using Equation (3) [39].

S% = [(W_s_ − W_d_)/W_d_] × 100 (1)

M% = [(W_s_ − W_d_)/W_s_] × 100 (2)

P% = [(W_s_ − W_sq_)/W_s_] × 100 (3)

Here, “W_s_”, “W_d_”, and “W_sq_” represent the weight of swollen, dry, and squeezed cryogel/composite, respectively.

To determine the pore volume (V_p_) of the cryogel composites, about 30 mg of cryogel/composite was weighed and these samples were kept in cyclohexane, a poor solvent for them, for 20 min, and reweighed. The V_p_ of the cryogel composites was calculated using Equation (4).

V_p_ = (W_ch_ − W_d_)/(W_d_ × d_ch_) (4)

Here, “W_ch_” is the weight of the cryogel/composite swollen in cyclohexane and “d_ch_” is the density of cyclohexane.

### 2.4. TA and Cur Release Profiles from PVA:TA and PVA:TA:Cur Cryogel Composites

Three different pH buffers were used to examine the TA release profile of PVA:TA cryogel composites, which differ in TA content by 1:0.1, 1:0.25, 1:0.5, and 1:1 by weight of PVA. Briefly, the TA release profiles from the PVA:TA cryogel composites were examined at 37.5 °C by weighing about 40 mg and placing it in 40 mL pH 1.0 (hydrochloric acid-potassium chloride buffer), pH 7.4 (phosphate buffer), and pH 9.0 (borate buffer) buffer solutions for 100 h. The amount of TA released was calculated from the previously prepared calibration curve for TA employing UV-Vis spectroscopy (UV-VIS Spectrophotometer, T80+, PG Inst., Ltd., Leicestershire, UK), at the maximum absorption wavelength of TA at 276 nm for pH 1.0 and pH 7.4, and at the wavelength of 296 nm for pH 9.0. For the samples, 1 mL was removed from the release medium and the absorbance values were recorded at the mentioned wavelengths and replaced with the corresponding fresh medium. Furthermore, TA release profiles from PVA:TA:Cur 1:1:0.1 cryogel composites obtained by adding Cur into PVA:TA cryogel composites were followed via UV-Vis spectroscopy, while the Cur release profiles were followed via fluorescence spectroscopy (Lumina, Seoul, Korea). In order to follow the Cur release from the PVA:TA:Cur 1:1:0.1 cryogel composites by fluorescence spectroscopy, primarily a calibration curve was constructed using EtOH and EtOH:Wat mixture at 528 nm emission wavelength and 500 V PMT voltage. Then, the amount of Cur released from the PVA:TA:Cur 1:1:0.1 cryogel composites was calculated using this calibration curve.

### 2.5. Hemocompatibility Testing for PVA Cryogel, PVA:TA, and PVA:TA:Cur Cryogel Composites

The hemocompatibility testing was performed in vitro to illustrate how the PVA cryogel, PVA:TA, and PVA:TA:Cur cryogel composites will affect red blood cells (hemolysis) and coagulation. The hemocompatibility tests of the PVA cryogel, PVA:TA, and PVA:TA:Cur cryogel composites were done in accordance with a procedure approved by Canakkale Onsekiz Mart University Clinical Research Ethics Committee (2011-KAEK-27/2020-E.2000045671). Details of hemocompatibility testing are elaborated in the Supporting Information.

### 2.6. Antioxidant Features of PVA:TA and PVA:TA:Cur Cryogel Composites

In medical/biomedical applications, it is very important that the material has antioxidant and antibacterial properties. The compounds containing phenolic groups are known to be effective antioxidant materials. The antioxidant activities of the PVA:TA and PVA:TA:Cur cryogel composites were determined using two different methods: TEAC and TPC.

#### 2.6.1. Total Phenol Content (TPC) Assay

The TPC assay was carried out for the samples taken from the TA release medium of the PVA:TA cryogel composites after 5 days. TA released from the PVA:TA cryogel composites (approximately 40 mg) in 40 mL of pH 7.4 buffer solution at 37.5 °C was used. Then, 0.1 mL of the release medium was transferred to a dark-colored bottle and 1.25 mL 0.2 N FC-phenol reagent solution was added. After 4 min, 0.7 M 1 mL Na_2_CO_3_ solution was added to the solution, and it was incubated for 2 h in the dark. Then, the absorbance measured for each specimen was determined via UV-Vis spectroscopy at a wavelength of 760 nm. The antioxidant activity of the PVA:TA:Cur cryogel composite was investigated by applying the same process with the samples withdrawn from the release medium of EtOH or EtOH:Wat at the concentration stated above. Gallic acid was used as a standard for the calculation of TPC.

#### 2.6.2. Trolox Equivalent Antioxidant (TEAC) Assay

To determine the TEAC value of the prepared PVA:TA and PVA:TA:Cur cryogel composites, the ABTS radical capture test was applied, similar to the method reported in the literature [7]. Here, the ABTS^.+^ radical was formed by the reaction of ABTS^.+^ and KPS. A solution containing 7.5 mL 7.00 mM ABTS and 2.5 mL 2.45 mM KPS was prepared and incubated for 12–16 h at room temperature in a dark environment to provide active radical formation. The prepared solution was used as a stock, and this solution was diluted to pH 7.4 until its absorbance at 734 nm was 0.7 ± 0.01 according to UV-Vis spectroscopy. Afterward, the specimens at various concentrations in the range of 0.0025–0.2 mL were added to 3 mL of ABTS^.+^ solution for an inhibition% value in the range of 20–80% from the equation stated below, and incubated for 6 min. The change in absorbance at 734 nm was followed. The ABTS^.+^ radical capture capacity was calculated using Equation (5).

Inhibition% = [(A_blank_ − A_specimen_)/A_blank_] × 100 (5)

where A_blank_ indicates the absorbance of the antioxidant-free ABTS^.+^ solution, and A_specimen_ is the absorbance of the ABTS^.+^ solution with the specimen. The values were plotted against Trolox standard curves, and the slope was determined. The TEAC values were calculated using the slope of the graphs. The TEAC values of the cryogel composites were expressed as “µmole trolox-equivalent/g specimen” (µmole TE/g).

### 2.7. Determination of Antimicrobial Effects of PVA Cryogel, PVA:TA, and PVA:TA:Cur Cryogel Composites

The antimicrobial activity of the PVA cryogel, PVA:TA, and PVA:TA:Cur cryogel composites was determined using the macro dilution method against Gram-negative *E. coli* and Gram-positive *S. aureus* microorganisms. Revival of bacterial colonies was done at room temperature and adjusted to McFarland 0.5 standard by suspending in nutrient broth at approximately 10^8^ CFU/mL (colony-forming unit). Before inoculation of bacterial strains, the PVA cryogel, PVA:TA, and PVA:TA:Cur cryogel composites were sterilized by keeping them under 420 nm UV light for 1 min.

#### Macro Dilution Method

Sterile PVA cryogel, PVA:TA, and PVA:TA:Cur cryogel composites in three different amounts of 50, 100, and 200 mg were added into 10 mL of nutrient broth. Then, 0.1 mL of microorganism suspension was added to the medium and incubated for 18–24 h in an oven at 37 °C. Then, 0.1 mL was withdrawn from the medium, and it was seeded in nutrient agar medium. If it was necessary, the broth media containing the bacteria was diluted with 0.9% saline solution. The next step was the incubation of seeded nutrient agars for 18–24 h in 35 °C ovens. The next day, the bacterial growth was detected by counting the colonies.

### 2.8. Enzymatic Assay

TA was dissolved at 1000 mg/mL in a pH 7.4 phosphate buffer. It was diluted to 500, 250, 125, 62.5, and 31.5 mg/mL with pH 7.4 PBS. In addition, 40 mg of the cryogels, PVA, PVA:TA 1:1, and PVA:TA:Cur, was put in 40 mL PBS and left for 2 days at 37.5 °C in a shaking bath to release the TA into the PBS. The release media was diluted to 500, 250, 125, and 62.5 µg/mL. Additionally, 40 mg PVA:TA:Cur cryogel was used in 40 mL EtOH:Wat (volume of 50:50). The effect of the TA and the TA-based cryogels on α-glucosidase (EC 3.2.1.20) was examined using the colorimetric substrate, p-nitrophenyl-α-D-glucoside, according to the literature with some minor modifications [40]. Briefly, 70 µL of different concentrations of TA or PVA:TA-based cryogel release media was put into the well plate with PBS as a control. An enzyme solution of 0.03 unit/mL in 70 µL was placed into the TA or TA release solution, and the mixed solution was read at 405 nm with a Thermo Scientific Multiskan Go microplate reader and incubated for 10 min. Finally, 10 Mm 70 µL substrate solution was put into the mixed solution. After 20 min, the absorbance of the mixed solution was measured. For the inhibition of the α-glucosidase, Equation (6) was used.

(6) $$\mathrm{Inhibition}\;\mathrm{of}\;\alpha -\mathrm{glucosidase}\;\mathrm{enzyme}\;\%=[(1-\frac{\Delta A_{405}^{sample}}{\Delta A_{405}^{control}})]\;\times \;100$$

where $\Delta A_{405}^{control}$ value is the change in absorbance value when the substrate is added to the control (PBS without sample) at 405 nm, and $\Delta A_{405}^{sample}$ is the change in absorbance value caused by adding the substrate to the sample solution.

## 3. Results

### 3.1. Synthesis and Characterization of PVA Cryogel, PVA:TA, and PVA:TA:Cur Cryogel Composites

**Figure 1:**
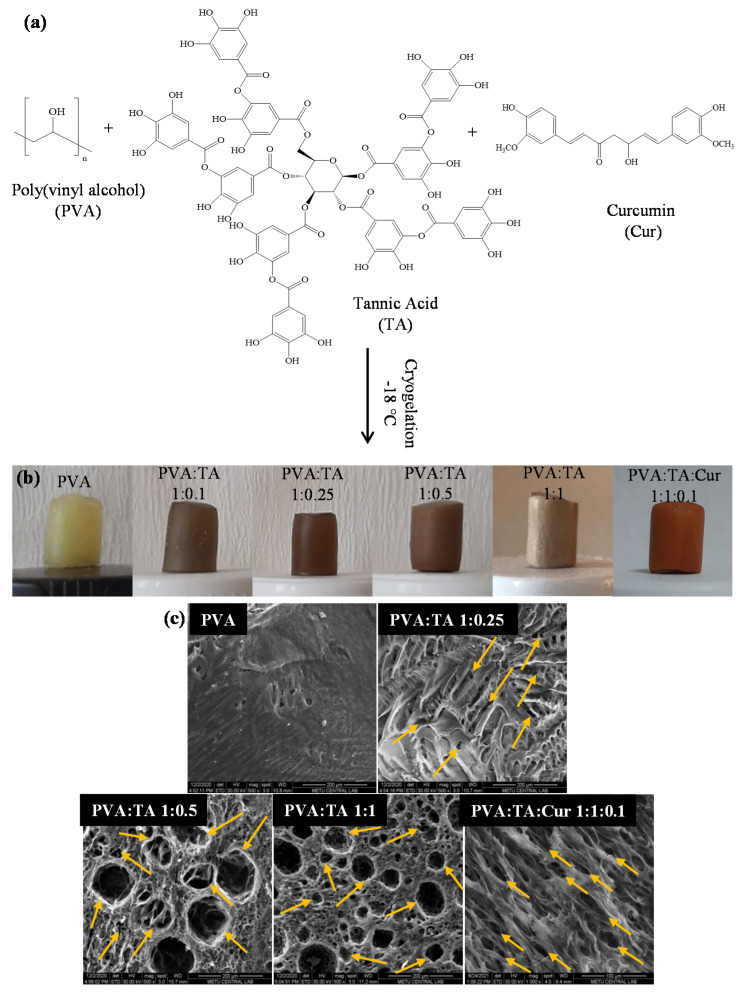
(**a**) Schematic representation, and (**b**) digital camera images of PVA cryogel, PVA-TA cryogel composites, and PVA-TA-Cur cryogel composites, and (**c**) their SEM images.

Cryogels are macroporous 3-D networks with customizable porosity, mechanical strength, and surface properties, and have been exploited in a variety of applications such as drug delivery, tissue engineering, and wound care applications, as well as in environmental remedies, e.g., organic and toxic waste removal and as chromatographic materials [1,2,3]. The PVA:TA and PVA:TA:Cur cryogel composites were synthesized using the cryogelation method in basic conditions, and the scheme for the preparation of the composite cryogel is illustrated in Figure 1a.

As can be seen from Figure 1b, physically crosslinked opaque brown PVA:TA and orangey PVA:TA:Cur cryogel composites were obtained by the formation of interpenetrated porous cryogel networks through freeze–thawing.

The SEM images of the PVA, PVA:TA 1:0.25, PVA:TA 1:0.5, PVA:TA 1:1, and PVA:TA:Cur 1:1:0.1 cryogel composites are given in Figure 1c. As seen in Figure 1c, while PVA cryogel networks have a non-porous, flat surface morphology, macro and spherelike porous structures with pore sizes ranging from 20–100 μm were obtained by the addition of different amounts of TA to the structure. The pore size of the PVA:TA and PVA:TA:Cur cryogel composites was determined by measuring 50 randomly selected pores from the SEM images using the Image J software program (Version, Company, City, Country). The pore sizes of the PVA:TA and PVA:TA:Cur cryogel composites were found to be in the 20 ± 4–100 ± 17 and 5 ± 1–50 ± 5 µm range, respectively. It is clear that with the addition of Cur to the PVA:TA cryogel composites, the pores of the cryogel composites became density packed and elongated. Furthermore, the pore sizes got smaller and changed from 20-–100 μm to 5–50 μm upon the addition of Cur into the cryogel network.

The S%, M%, and P% of the PVA cryogel, PVA:TA, and PVA:TA:Cur cryogel composites were calculated from the swelling studies in pH 7.4 buffer solutions, and the results are summarized in Table 1. As seen in Table 1, the calculated maximum swelling capacity for the PVA cryogel is 169 ± 6%, and the PVA:TA 1:1 cryogel composite had a maximum swelling capacity of 258 ± 62%. The swelling capacities of cryogel composites containing PVA:TA increased along with the increase in the TA content. This is reasonable, as an increase in amount of TA increases the number of hydroxyl groups that eventually increase the hydrophilicity of the cryogel composite. The values for the other materials are between these two limits. The M% values for the PVA cryogel and the PVA:TA 1:1 cryogel composite were calculated as 63 ± 2% and 72 ± 6%, respectively. Moreover, the M% values for the other materials are also between these two limits. Nevertheless, it is clear that there is no considerable difference for the M% values of all of the prepared samples. The P% of the PVA cryogel, PVA:TA 1:0.1, PVA:TA 1:1:0.25, PVA:TA 1:0.5, PVA:TA 1:1, and PVA:TA:Cur 1:1:0.1 cryogel composites were calculated as 8 ± 4%, 10 ± 3%, 10% ± 1%, 17 ± 1, 43 ± 5%, and 38 ± 5%, respectively. The SEM images also support the P% results. As the amount of TA in the PVA-based cryogel/composite increased, the P% of the cryogel composites also increased, and the presence of Cur in the PVA:TA:Cur 1:1:0.1 cryogel composites slightly decreased the P% value of PVA:TA 1:1.

The pore volume (V_p_) of the PVA cryogel was calculated as 0.1 ± 0.05% and for the PVA:TA 1:1 cryogel/composite was 0.69 ± 0.32%. The results for other cryogel composites are within these two limits, and for the V_p_ values, very similar trends to the P% values were observed.

**Figure 2:**
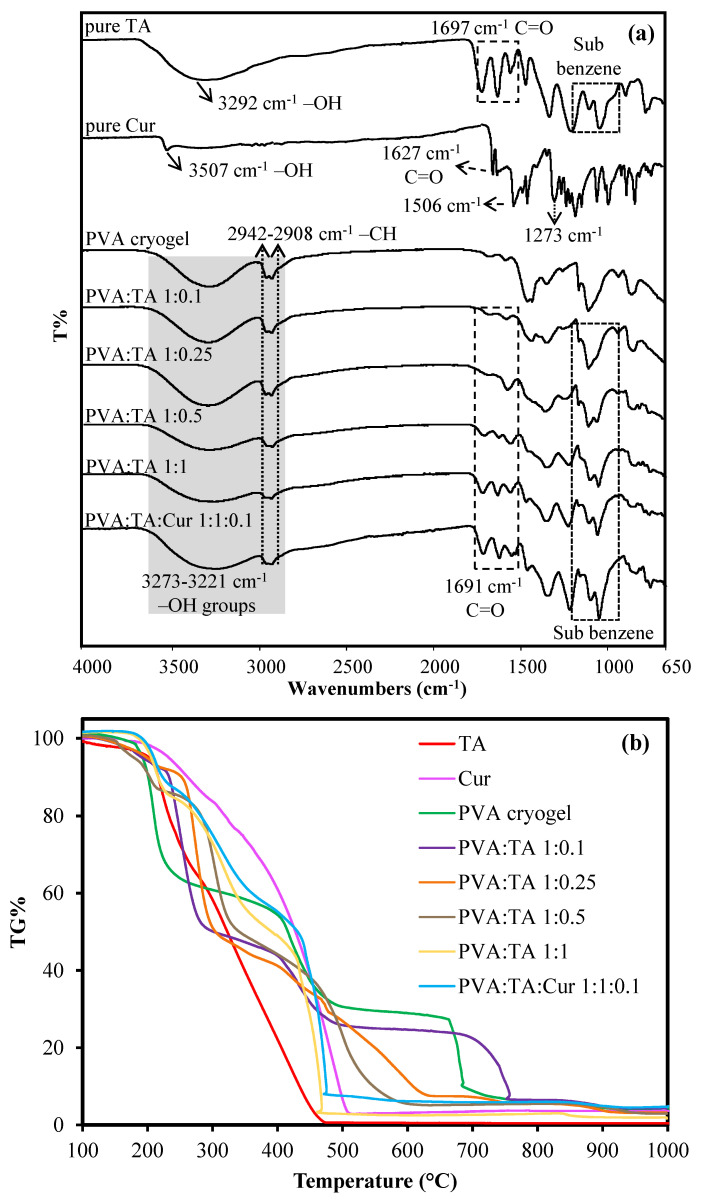
(**a**) FTIR spectra and (**b**) TG thermograms of pure TA, pure Cur, PVA cryogel, PVA:TA, and PVA:TA:Cur cryogel composites.

The structural and thermal characterization of pure TA, pure Cur, PVA cryogel, PVA:TA, and PVA:TA:Cur cryogel composites were performed using FT-IR and TGA analysis, respectively, and the related results are given in Figure 2a,b, respectively. Characteristic bands of pure TA as –OH band and C=O stretchings were observed at 3292 and 1697 cm^−1^, respectively. Other characteristic bands for pure TA corresponding to the aromatic group and substituted benzenes were found between 1435–1607 cm^−1^ and 1079–1309 cm^−1^, respectively [11,41]. On the other hand, the –OH stretching of phenol, C=O, and C=C bands were also observed at 3507, 1627, and 1506 cm^−1^ as the characteristic bands of pure Cur, respectively. Moreover, the C-O stretching bands of the ether groups were observed at 1273 cm^−1^, whereas the bands at 961, 808, and 714 cm^−1^ were assigned to C-H bending [35,42].

As the FT-IR spectra of the cryogels given in Figure 2a are examined, characteristic bands such as -OH band at 3272 cm^−1^ and -CH bands at 1563, 1437, and 1327 cm^−1^ were observed for PVA. After cryogelation of the PVA with TA, the characteristic bands of PVA remain unchanged, while a C=O characteristic band at 1691 cm^−1^ arising from TA and characteristic aromatic groups appeared at 1609, 1535, and 1445 cm^−1^. In addition, the characteristic bands at 1080–1200 cm^−1^ are ascribed to the sub benzene group, also originating from the TA. Moreover, the band at 2942–2908 cm^−1^ for methylene attributed to methylene groups was also maintained and widened with inclusion of TA into the structure. In addition to all of these bands in the FT-IR spectrum of the PVA:TA:Cur 1:1:0.1 cryogel/composite, new bands from C-O-C stretching originating from Cur were observed at 960 and 910 cm^−1^. As the amount of TA increased, the O-H absorption band (3300–3272) weakened, and the location of the band shifted. Hydrogen bonding interactions occur between TA and PVA. The intra- and inter hydrogen bonding within and amongst the TA molecules, as well as between TA and PVA, overlaps with OH band 3273–3570 cm^−1^ [43]. It is apparent from the FT-IR spectra of the PVA:TA cryogel composites that as the amount of TA increased, the -OH bands became wider. These observations are in accordance with the reports in the literature regarding structures containing PVA and polyphenol [44,45]. The literature is compatible with the characterization results.

The thermal characterization of pure TA, pure Cur, PVA cryogel, PVA:TA, and PVA:TA:Cur cryogel composite was determined via TG analyzer, and the relevant thermograms are illustrated in Figure 2b. In addition, the temperatures corresponding to 5% and 10% weight loss of samples are summarized in Table S1. As can be seen in Table S1, the samples showed 5% weight loss at temperatures between 104 and 233 °C. On the other hand, the same samples showed 10% weight loss at temperatures between 194 and 252 °C. Pure TA begins to degrade at 104–203 °C with a 5% weight loss, and at 470 °C more than 99% of its weight is degraded. Nevertheless, pure Cur starts to degrade at 169–233 °C with a 5% weight loss and is almost completely degraded at 510 °C with a weight loss of 97%. On the other hand, the PVA cryogel and PVA:TA 1:0.1 had similar thermal degradation profiles because the PVA:TA 1: 0.1 cryogel/composite contains a small amount of TA. The PVA cryogel and PVA:TA 1:0.1 cryogel/composite were degraded in three steps. The first degradation step for the PVA cryogel and PVA:TA 1:0.1 cryogel composite were observed between 167–224 °C and 122–147 °C, respectively, and these correspond to 2–4% weight loss which is due to the loss of the absorbed water molecules. For the PVA cryogel and PVA:TA 1:0.1 cryogel composite, the second degradation steps occurred between 225–377 and 143–230 °C, respectively, and these are also associated with the loss of the bound water to the polymer matrix. Both materials showed a multi-step weight loss of about 60–62%, and the last degradation steps were observed between 480–1000 °C and 490–1000 °C due to the carbonization of the polymer. The last stages ended with a weight loss of 96% for both materials. Apart from these, the PVA:TA1:0.25, PVA:TA 1:0.5, PVA:TA 1:1, and PVA:TA:Cur 1:1:0.1 cryogel composites also exhibited similar thermal degradation profiles. These cryogel composites were degraded in two stages. The PVA:TA 1:0.25, PVA:TA 1:0.5, PVA:TA 1:1, and PVA:TA:Cur 1:1:0.1 cryogel composites started to degrade at 142, 190, 198, and 198 °C, respectively, with a weight loss of approximately 2–4%. The first stage of degradation started between 142 and 198 °C for these cryogels, and the weight losses observed in the first stage are due to the removal of moisture and water molecules [46]. The second stages started between 460 and 480 °C, and the final weight loss resulted in 95–98% weight loss at 1000 °C for all cryogel composites. The degradations observed in the second stage at the temperature range of 230–400 °C are attributed to the decarboxylation of the gallic acid unit [47]. The thermograms show that the cryogel composites are thermally slightly more stable than the PVA cryogel, and thermal degradations started at early temperatures with an increase in the amount of TA within the cryogel composites. The presence of Cur also strongly effected the early thermal degradation of the PVA:TA cryogel composites by increasing the early thermal degradation to higher temperatures in comparison to the PVA:TA cryogel composites; however, at about T > 420 °C, no significant thermal degradation difference was observed between PVA:TA 1:1: and PVA:TA:Cur 1:1:0.1.

### 3.2. TA and Cur Release from Cryogel/Composite Matrices

**Figure 3:**
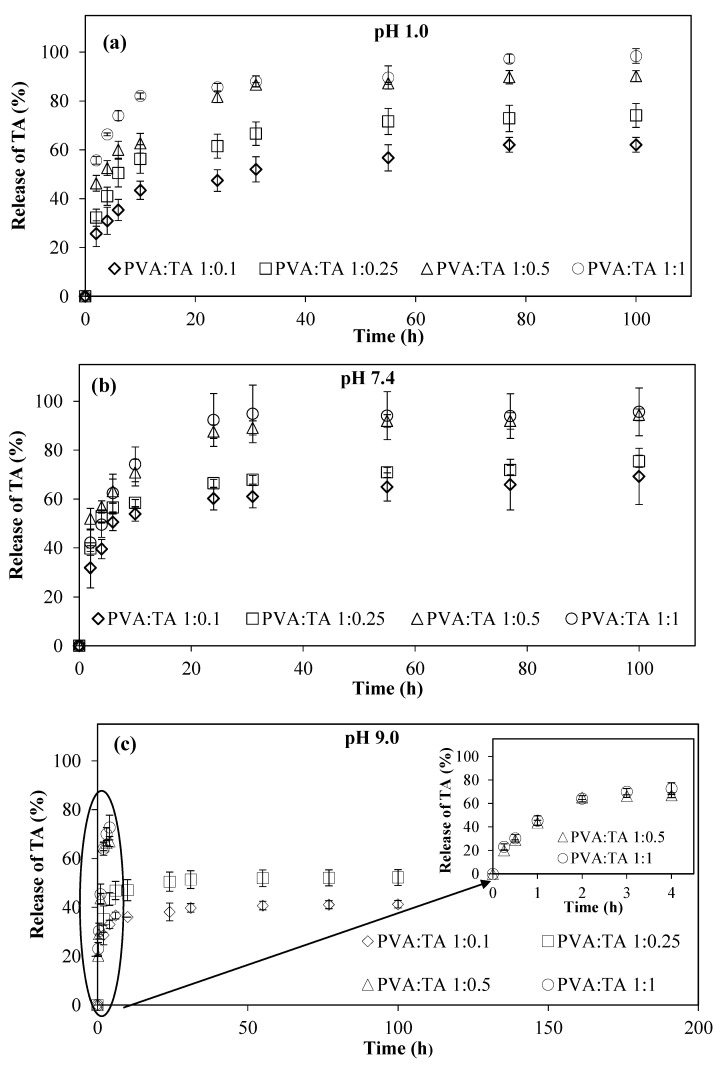
TA release profiles from PVA:TA cryogel composites at (**a**) pH 1.0, (**b**) pH 7.4, and (**c**) pH 9.0.

There is a growing interest in materials containing TA for pharmaceutical and biomedical applications due to their effective antioxidant, anti-bacterial, and anti-inflammatory effects. Therefore, PVA:TA cryogel composites can be used as drug or bioactive molecule carrier and delivery systems, e.g., TA release systems. Different parts of the human body have different pH values, e.g., the pH of the stomach is about pH 1.0, blood plasma (physiological) is pH 7.4, and the pancreatic and intestinal pH values are about 9.0. Therefore, the release studies from the PVA:TA cryogel composites were investigated at these three different pH conditions to demonstrate their potential biomedical usability. Three different pH buffer solutions of pH 1.0, pH 7.4, and pH 9.0 were used to examine TA release from the PVA:TA cryogel/composite matrices prepared at a weight ratio of 1:0.1, 1:0.25, 1:0.5, and 1:1. The amount of TA released was detected by taking specimens from the release medium at certain time intervals up to 100 h of release, and then measuring the absorbance values of the solutions using UV-Vis spectroscopy. Firstly, to calculate the TA loading amount of the cryogel composites, approximately 40 mg of sample was placed in a 40 mL of pH 9 buffer solution. Then, the sample-containing tubes were placed at 80 °C for complete degradation of the cryogel composites. Since the degraded cryogels will release all of the TA within, the absorbances were measured from the previously constructed calibration curves, as mentioned in the release studies, and the amount of TA loaded was calculated. The amount of TA within the PVA:TA cryogel composites was calculated as 98 ± 27, 218 ± 41, 413 ± 48, and 535 ± 87 mg/g for PVA:TA 1:0.1, PVA:TA 1:0.25, PVA:TA 1:0.5, and PVA:TA 1:1 cryogel composites, respectively. In Figure 3, the released% of TA from each cryogel matrix for each pH buffer solution is given. As can be clearly seen from Figure 3, the released% of TA from each cryogel matrix increases with the increase in the amount of TA present in the cryogel composites. All cryogel composites, except PVA:TA 1:0.5 and PVA:TA 1:1, released TA linearly for up to 10 h at pH 1.0, pH 7.4, and pH 9.0. The TA release results from the PVA:TA 1:0.1, PVA:TA 1:0.25, PVA:TA 1:0.5, and PVA:TA 1:1 cryogel composites at pH 1.0 are given in Figure 3a. These cryogel composites released TA from the cryogel matrices in a linear fashion for up to 10 h. After 100 h, 62 ± 3, 74 ± 5, 90 ± 2, and 98 ± 3% of TA was released from the PVA:TA 1:0.1, PVA:TA 1:0.25, PVA:TA 1:0.5, and PVA:TA 1:1 cryogel composites, respectively. According to the results shown in Figure 3b, within 10 h, the PVA:TA 1:0.1, PVA:TA 1:0.25, PVA:TA 1:0.5, and PVA:TA 1:1 cryogel composites had released 53 ± 4, 58 ± 2, 70 ± 5, and 74 ± 7% of TA at pH 7.4, respectively. After 100 h, the PVA:TA 1:0.1, PVA:TA 1:0.25, PVA:TA 1:0.5 and PVA:TA 1:1 cryogel composites had released 69 ± 11, 75 ± 3, 94 ± 2, and 96 ± 10% of TA, respectively.

Finally, the amount of TA released from the PVA:TA 1:0.1, PVA:TA 1:0.25, PVA:TA 1:0.5, and PVA:TA 1:1 cryogel composites at pH 9.0 is given in Figure 3c. The PVA:TA 1:0.1 and PVA:TA 1:0.25 cryogel composites released 36 ± 1 and 47 ± 5% of the TA in the first 10 h. These cryogel composites released 41 ± 2 and 52 ± 3% TA after 100 h. Interestingly, the PVA:TA 1:0.5 and PVA:TA 1:1 cryogel composites given in the Figure 3c inset completely degraded within 4 h, and had released 67 ± 2 and 73 ± 5% TA. As a result, as shown in Figure 3, the release rates depend on the amounts of TA added into the cryogel composites during synthesis and the pH conditions of medium. The amount and rate of TA release increased with increasing TA amount in the cryogel composites, as anticipated. This provides a great advantage for designing a controllable release profile for TA. The PVA:TA cryogel composites released higher amounts of TA under neutral conditions than under acidic and basic conditions.

Apart from these, the TA and Cur release profiles in EtOH and EtOH:Wat (1:1 by volume) mixture from the PVA:TA:Cur 1:1:0.1 cryogel composites were investigated. Since Cur is a hydrophobic material, the release studies were also carried out in EtOH and EtOH:Wat, where the solubility of Cur is higher. While the TA release profile of the PVA:TA:Cur 1:1:0.1 cryogel composites was followed via UV-Vis spectroscopy, the release profile of Cur, a fluorescent material, was followed via fluorescence spectroscopy.

**Figure 4:**
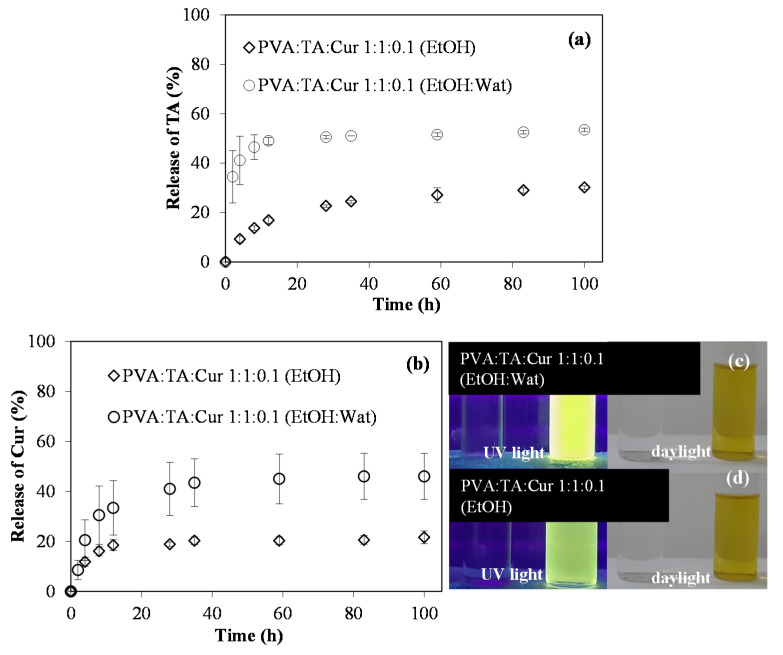
TA and Cur release profiles from PVA:TA:Cur 1:1:0.1 cryogel composites, (**a**) TA release profiles in EtOH and EtOH:Wat by UV-Vis spectroscopy, and (**b**) Cur release profiles in EtOH and EtOH:Wat by fluorescence spectroscopy. Digital camera images of these cryogels under (**c**) 366 nm UV light, and (**d**) daylight.

In Figure 4a, the amounts of TA released from the PVA:TA:Cur 1:1:0.1 cryogel composites are given. The release was monitored in EtOH and EtOH:Wat media and examined with UV-Vis spectroscopy. According to the release amounts presented in the graphics, the PVA:TA:Cur 1:1:0.1 cryogel composites released 30 ± 1% TA in EtOH and 54 ± 1% TA in the EtOH:Wat mixture in 100 h. On the other hand, the amounts of Cur released from the PVA:TA:Cur 1:1:0.1 cryogel composite in EtOH and EtOH:Wat media, which were assessed via fluorescence spectroscopy, are given in Figure 4b. These cryogels/composites released 22 ± 5 and 46 ± 9% of Cur within 100 h. In Figure 4c, and d, the digital camera images of the samples drawn from the EtOH and EtOH:Wat mixture of these cryogel composites are given under 366 nm UV light and daylight. While the samples displayed a yellowish green appearance in daylight, they have a bright yellow-green appearance under UV light due to the fluorescent nature of the Cur molecules. As can be seen from both the release results and the digital camera images, the PVA:TA:Cur 1:1:0.1 cryogel composites had a higher release performance in the EtOH:Wat medium. As a result, the PVA:TA:Cur 1:1:0.1 cryogel composites showed the highest release profile not only for Cur, but also TA in the EtOH:Wat medium.

### 3.3. Hemocompatibility Test

**Figure 5:**
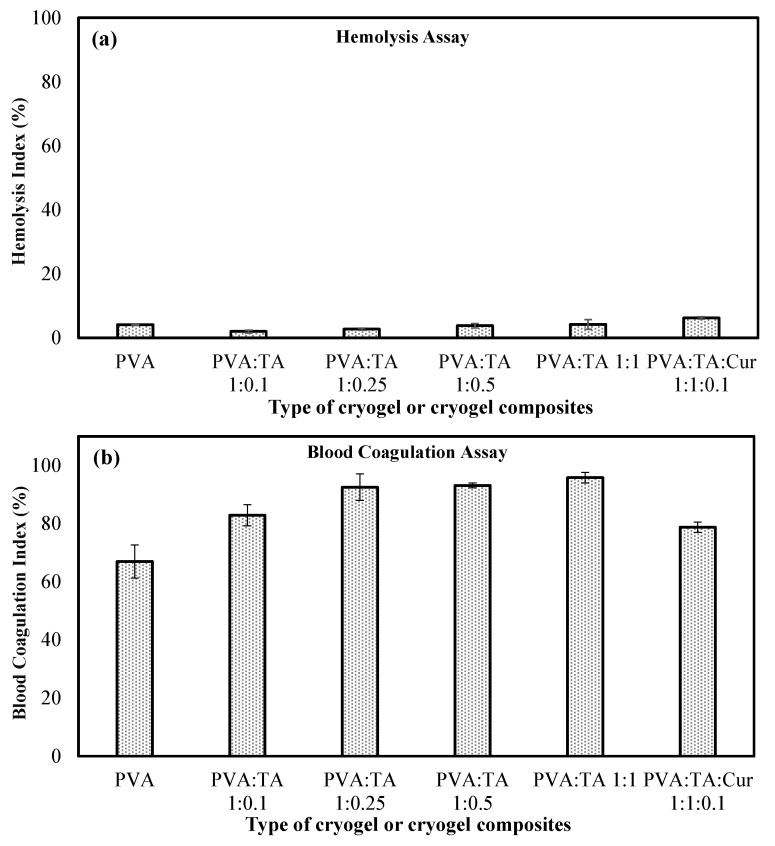
Hemocompatibility of PVA cryogel, PVA:TA, and PVA:TA:Cur cryogel composites according to (**a**) hemolysis assay and (**b**) blood coagulation assay.

In cases where materials encounter blood, it is expected that the materials will not change blood parameters and cause unwanted side effects. Hemolysis is the destruction of erythrocytes (red blood cells), and blood compatibility is classified as non-hemolytic between 0 and 2%, less hemolytic between 2 and 5%, and hemolytic at >5%, according to hemolysis rates. Blood compatibility tests were carried out in order to define the potential use of PVA cryogel and cryogel composites, which can come into contact with blood in various biomedical applications in the medical/biomedical field. Therefore, the blood compatibility tests of the materials were determined by the hemolysis% and blood coagulation% test. The hemolysis and blood coagulation test results for the blood compatibility of the PVA cryogel, PVA:TA 1:0.25, PVA:TA 1:0.5, PVA:TA 1:1, and PVA:TA:Cur 1:1:0.1 cryogel composites are given in Figure 5a,b, respectively.

From the hemolysis test results, the PVA cryogel and PVA:TA cryogel composites have slight hemolytic effects, with hemolysis rates of 4.10 ± 0.10%, 2.00 ± 0.43%, 2.70 ± 0.35%, 3.81 ± 0.73%, and 4.20 ± 1.53%, respectively. On the other hand, the PVA:TA:Cur cryogel/composite shows little hemolytic effect, with a hemolysis rate of 6.22 ± 0.40% after contact with blood. When examining the blood coagulation test results, the PVA cryogel, PVA:TA cryogel composites, and PVA:TA:Cur cryogel composite have blood coagulation indices of 67 ± 6%, 83 ± 4%, 93 ± 5%, 93 ± 1%, 96 ± 2%, and 79 ± 2%, respectively. It is clear that none of the PVA:TA cryogel composites affect the blood coagulation mechanism, but the PVA cryogel and PVA:TA:Cur 1:1:0.1 cryogel/composite do have some coagulating effects. As a result, irrespective of the PVA cryogel and the amount of TA it contains, PVA:TA cryogel composites are materials with little hemolytic effect and the PVA:TA:Cur 1:1:0.1 cryogel composite is a material with a hemolytic effect.

### 3.4. Antioxidant Features

In medical/biomedical applications, the antioxidant and antibacterial properties of the material are also very important. Antioxidant materials can have synthetic and natural components. However, since synthetic antioxidants can render additional negative effects on health, research about natural antioxidants has been gaining significant importance recently. [48]. Natural antioxidants interact with and neutralize free radicals that are the culprits for the many health related diseases. Thus, they play an important role in the treatment of oxidative stress-induced diseases caused by free radicals. Phenol-containing compounds are effective antioxidants and protect living cells and proteins from the damaging effects of free radicals [49,50,51].

The antioxidant properties of the PVA:TA and PVA:TA:Cur cryogel composites in pH 7.4 buffer solution, EtOH, and EtOH:Wat were determined using two different antioxidant methods—TPC and TEAC—and the results are summarized in Table 2.

The antioxidant equivalent capacity of the PVA:TA 1:0.1, PVA:TA 1:0.25, PVA:TA 1:0.5, and PVA:TA 1:1 cryogel composites in pH 7.4 were calculated as 5.14 ± 0.26 µg/mL, 18.45 ± 0.13 µg/mL, 54.62 ± 1.33 µg/mL, and 65.28 ± 0.11 µg/mL gallic acid equivalent, respectively. The antioxidant equivalent capacity of the PVA:TA:Cur 1:1:0 cryogel composites in EtOH and EtOH:Wat was calculated as 235.41 ± 4.00 µg/mL and 292.71 ± 11.50 µg/mL gallic acid equivalent, respectively. The TEAC values for the PVA:TA 1:0.1, PVA:TA 1:0.25, PVA:TA 1:0.5, and PVA:TA 1:1 cryogel composites were calculated as 0.06 ± 0.01, 0.11 ± 0.01, 0.17 ± 0.05, and 0.75 ± 0.01 µmole TE/g, respectively. The TEAC values for the PVA:TA:Cur 1:1:0.1 cryogel composites in EtOH and EtOH:Wat mixture were calculated as 2.01 ± 0.22 and 2.10 ± 0.24 µmole TE/g, respectively. Both antioxidant test results affirm that the PVA:TA and PVA:TA:Cur cryogel composites have prominent antioxidant traits that can be useful in different bio-medicinal applications.

### 3.5. Antimicrobial Activity

The lowest concentration with no visual growth in nutrient broth after incubation is determined as the minimum inhibition concentration (MIC) value. Furthermore, the lowest concentration value where there is no growth—in other words, the number of organisms decreased by >99.99%—is accepted as the minimum bactericidal concentration (MBC). The antimicrobial activities of the PVA:TA and PVA:TA:Cur cryogel composites were determined using the macro dilution method against *E. coli* and *S. aureus* microorganisms, and the corresponding results are summarized in Table 3. According to the results, the PVA:TA 1:0.1 and PVA:TA 1:0.25 cryogel composites exhibited no antimicrobial activity on either of the microorganisms, while the PVA:TA 1:0.5 and PVA:TA 1:1 cryogel composites both attained MIC values at 10 mg/mL concentration, and the MBC values were found to be at 20 mg/mL concentration against *E. coli*. The MIC values for the PVA:TA 1:0.5 and PVA:TA 1:1 cryogel composites against *S. aureus* were detected at 10 mg/mL and 5 mg/mL, respectively, and the MBC values for both cryogel composites were found at 20 mg/mL concentration.

As seen in Table 3, increasing amounts of TA in the cryogels elevated the anti-bacterial activity against both Gram-negative and Gram-positive bacteria. Nevertheless, the antibacterial outcomes between the PVA:TA 1:0.5 and PVA:TA 1:1 cryogels were observed to be similar, except that PVA:TA 1:1 was ascertained to be more effective on *S. aureus*.

**Figure 6:**
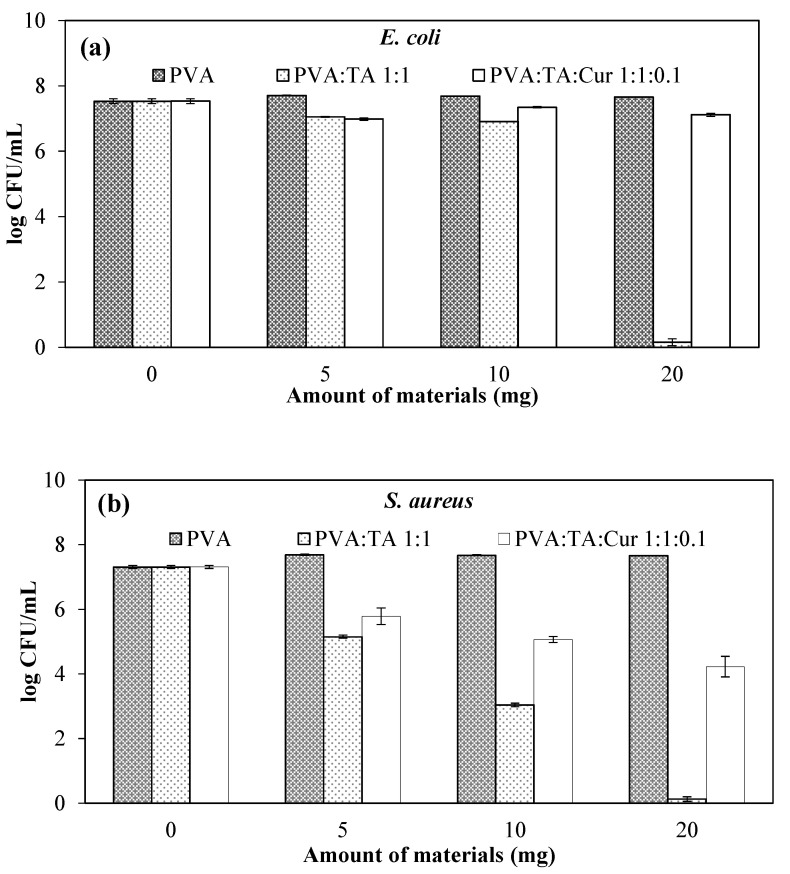
Bacterial growth number (log colony forming unit ((CFU)/mL)) in nutrient broth in the presence of PVA:TA:Cur 1:1:0.1 cryogel composites for (**a**) Gram-negative *E. coli* and (**b**) Gram-positive *S. aureus*.

Cur is a hydrophobic material and suppresses the release of TA. Therefore, the antimicrobial activities of PVA:TA:Cur cryogels containing Cur could not be calculated as MIC and MBC values. As a control, numbers of bacterial colonies (log CFU/mL) in nutrient broth were compared with nutrient broth containing various concentrations of PVA:TA:Cur 1:1:0:1 cryogel composites (such as 5, 10, and 20 mg/mL). The results are given for Gram-negative *E. coli* and Gram-positive *S. aureus* bacteria in Figure 6a,b as the number of bacteria colonies, respectively. As can be seen from the results in Figure 6a, none of the PVA:TA:Cur 1:1:0.1 cryogel composites dramatically affected the growth of *E. coli*, regardless of the amount compared to control. The growth of bacterial colonies only decreased from 10^7^ to 10^6^ CFU/mL relative to the control, which is approximately the same as the control group.

For *S.aureus* bacteria, 10^1^ CFU/mL bacteria formation was observed in the medium containing PVA:TA 1:1 cryogel/composite, while 10^6^ CFU/mL bacteria formed in the control group. Interestingly, 10^3^ CFU/mL bacterial colonies were detected in the medium containing 20 mg/mL PVA:TA:Cur 1:1:0.1 cryogel/composite. The PVA:TA 1:1 cryogel/composite used at maximum concentration exhibited high antimicrobial activity for both bacterial groups. In particular, the PVA:TA 1:1 cryogel/composite was found to be more effective on the *S. aureus* microorganism than the *E. coli* microorganism. These results revealed that the addition of Cur molecules to the cryogel composites decreased the antimicrobial activity and decreased the release of TA due to the hydrophobic property of Cur, but it created almost 50% inhibition for the *S. aureus* microorganism. As a result, the antimicrobial activities of the PVA:TA cryogel composites can be controlled depending on the bacterial species and the amount used. Therefore, the synthesized cryogels can be used in applications where antimicrobial and antioxidant properties are desired together.

### 3.6. Enzymatic Assay

**Figure 7:**
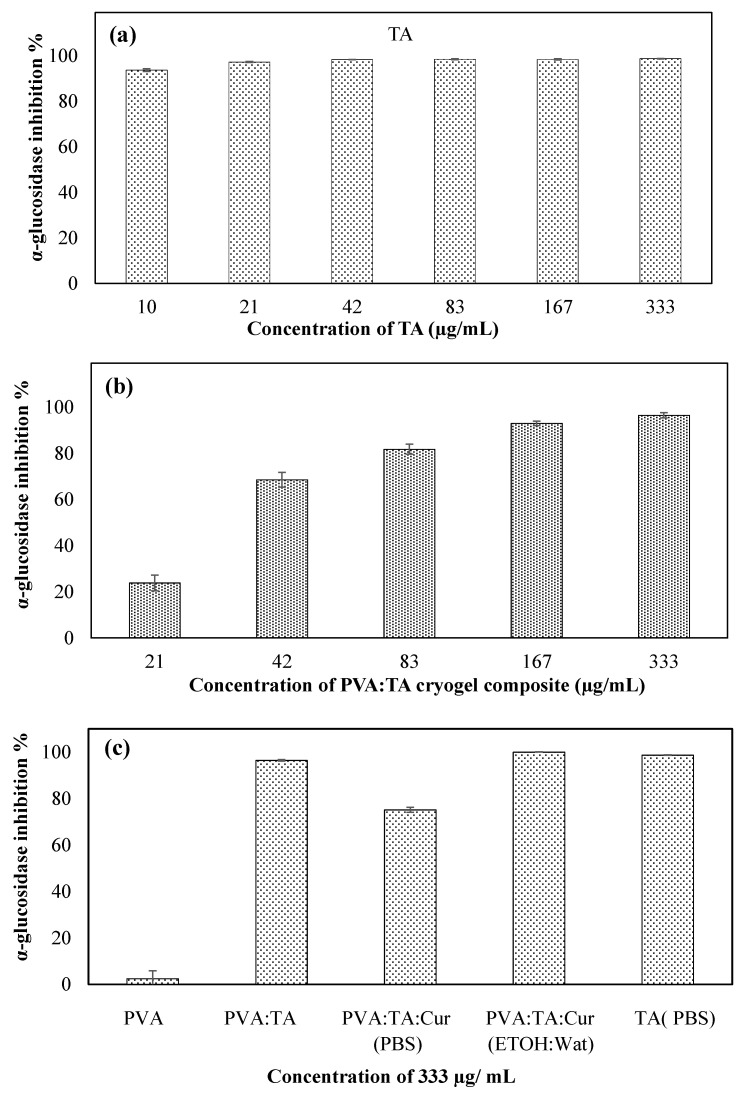
The α-glucosidase inhibitory activity of (**a**) TA, (**b**) PVA:TA cryogel composites at pH 7.4 PBS (different concentrations; 333, 167, 83, 42, and 21 μg/mL). (**c**) The α-glucosidase inhibitory activity of PVA:TA cryogel composites at pH 7.4 (at 333 µg/mL concentration).

The enzyme inhibition studies of the PVA:TA cryogels containing different amounts of TA are given in Figure 7a–c. As can be seen in Figure 7a, TA inhibits the alpha enzyme at high rates even at very low concentrations; e.g., even with a concentration of 10 µg/mL, it inhibits the enzyme activity by 93.71 ± 0.58%.

In Figure 7b, enzyme inhibition is given depending on the concentration of PVA:TA cryogel in the media. As the concentration of PVA:TA in media increases, the enzyme inhibition also increases. For 333 µg/mL PVA:TA cryogel, 96.41 ± 0.34% inhibition was observed. In Figure 7c, enzyme inhibition is given for 333 µg/mL of the PVA:TA-based cryogels. While the inhibition of the PVA:TA:Cur cryogels in PBS medium was 75.1 ± 1.03%, it was found to be 99.97 ± 0.04% in the EtOH:Wat mixture. This result is due to the dissolution of curcumin in EtOH:Wat medium. The TA contained in the cryogels did not lose any of its activity when the PVA:TA cryogels were synthesized.

## 4. Discussion

Here, PVA, a non-toxic, biocompatible, and non-hemolytic synthetic polymer [52] with high mechanical strength and viscoelastic properties was used in cryogel synthesis with different amounts of TA and Cur. The morphological structure of the cryogel and swelling studies revealed that the presence of TA, added during synthesis, changed the color, swelling capacity, pore percentage, and pore volume, which were found to increase along with an increase in the amount of TA. In the TA release studies, it was determined that the PVA:TA 1:1 cryogel released 92.3% of TA linearly for 24 h in PBS medium. Based on the pores formed depending on the amount of TA and the high release in the first 24 h, it is assumed that TA does not bind to PVA chemically or interact with PVA physically. The porosity of the cryogel composites can be adjusted by the control of TA amount used during synthesis. The porosity is one of the very important parameters for implant or graft materials, as it affects the adhesion and proliferation of the cells [53].

Because of the presence of polyphenolic TA and Cur, the prepared PVA:TA and PVA:TA:Cur cryogel composites possess bioactive properties. It was found that all PVA:TA cryogel composites have an insignificant effect on the blood coagulation mechanism, whereas the PVA cryogel and PVA:TA:Cur 1:1:0.1 cryogel composite possess some coagulating effects. Furthermore, it was illustrated that the synthesis of cryogels with antioxidant, antibacterial, and α-glycosidase inhibition properties is now possible, and can be readily controlled with use of appropriate amounts of TA and Cur. It is known that polyphenols facilitate intracellular interactions by modifying the hardness of the bacterial cell wall and binding hydrogen to enzymes around the cells, thus exhibiting antibacterial effects [54,55]. It was observed that the binding of polyphenols to enzymes directly affects the activity of the enzymes, as well as affecting the antimicrobial properties. It was observed that α-glycosidase enzyme inhibition by PVA:TA concentration is TA-dependent and increases with an increase in the TA content of PVA:TA cryogel composites.

It is known that a fibrin coating at the anastomosis of a vascular graft will greatly facilitate the healing [52]. TA influences the structure and conformation of fibrinogen to form fibrin [56]. Therefore, PVA:TA cryogels may provide a fibrin coating for artificial vein applications. Additionally, it is reported that PVA hydrogels are suitable for tissue engineering applications involving various tissues, such as skin/wound, bone, cartilage, vascular, cardiac, cardiovascular tissue, meniscus, human prostate tissue, and artificial corneas [52,57]. Therefore, according to the results of this investigation, PVA:TA cryogels have great potential for vascular and cardiovascular applications.

## 5. Conclusions

The present paper reports the successful preparation of PVA cryogel, PVA:TA, and PVA:TA:Cur cryogel composites. By assessing the TA and Cur release profiles of the prepared cryogel composites using both UV-Vis and fluorescence spectroscopy, a versatile material was developed that can release TA in a controlled manner along with Cur. PVA:TA and PVA:TA:Cur cryogel composites with desired bioactivity, such as blood compatibility and/or blood coagulation abilities as well as antioxidant and antibacterial properties, can be readily attained. PVA:TA, a superporous material with enhanced mechanical properties, is a promising candidate as a multifunctional agent for a variety of biomedical applications. PVA:TA cryogels possess a high blood clotting index value, and the coagulant effect of cryogels containing TA is lower than that of PVA. It is known that TA promotes the coagulation effect [58,59]. As illuminated here, TA that has many hydroxyl groups can readily interact with PVA via hydrogen bonding, affording a lower blood clotting index capability than PVA cryogels and thus promoting the blood contacting application of the prepared materials. In other words, TA inhibits PVA cryogels’ hemostatic properties. As PVA:TA cryogels do not cause coagulation, these materials can be suitable for many biomedical applications, including artificial veins. Furthermore, this type of cryogel composite can also be used as an enzyme inhibition material, as it was observed that the presence of TA in a cryogel composite results in concentration-dependent α-glycosidase enzyme inhibition capabilities. The reinforcement of nontoxic, biocompatible synthetic PVA polymers with highly antioxidant natural polyphenol TA and Cur is anticipated to enable the design of extraordinary prospective templates for a wide range of biomedical applications. Future studies with regard to the use of the prepared cryogel composite will be focused on various cells and tissues of animals, as well on in vivo studies.

## Figures and Tables

**Table 1:** Swelling rate (S%), moisture content (M%), porosity (P%), and total pore volume (V_p_) of PVA cryogel, PVA:TA 1:0.1, PVA:TA 1:1:0.25, PVA:TA 1:0.5, PVA:TA 1:1, and PVA:TA:Cur 1:1:0.1 cryogel composites.

Materials	S%	M%	P%	V_p_ (mL/g)
PVA	169 ± 6	63 ± 2	8 ± 4	0.10 ± 0.05
PVA:TA 1:0.1	198 ± 17	66 ± 2	10 ± 3	0.13 ± 0.06
PVA:TA 1:0.25	239 ± 23	71 ± 2	10 ± 1	0.19 ± 0.07
PVA:TA 1:0.5	239 ± 91	71 ± 9	17 ± 1	0.27 ± 0.17
PVA:TA 1:1	258 ± 62	72 ± 6	43 ± 5	0.69 ± 0.32
PVA:TA:Cur 1:1:0.1	229 ± 31	69 ± 3	38 ± 5	0.66 ± 0.19

**Table 2:** Antioxidant activity of PVA:TA and PVA:TA:Cur cryogel composites; TPC value and TEAC.

Release Conditions	Antioxidant Materials	TPC (µg/mL)	TEAC (µmole TE/g)
pH 7.4	PVA:TA 1:0.1	5.14 ± 0.26	0.06 ± 0.01
	PVA:TA 1:0.25	18.45 ± 0.13	0.11 ± 0.01
	PVA:TA 1:0.5	54.62 ± 1.33	0.17 ± 0.05
	PVA:TA 1:1	65.28 ± 0.11	0.75 ± 0.01
EtOH	PVA:TA:Cur 1:1:0.1	235.41 ± 4.00	2.01 ± 0.22
EtOH:Wat	PVA:TA:Cur 1:1:0.1	292.71 ± 11.50	2.10 ± 0.24

**Table 3:** Minimum inhibition concentration (MIC) and minimum bactericidal concentration (MBC) values of PVA cryogels at a single concentration and PVA:TA cryogel composites at different amounts against *E. coli* and *S. aureus* strains.

Organisms	*E. coli*		*S. aureus*	
Antimicrobial Materials	MIC (mg/mL)	MBC (mg/mL)	MBC (mg/mL)	MIC (mg/mL)
PVA	N.D.	N.D.	N.D.	N.D.
PVA:TA 1:0.1	N.D.	N.D.	N.D.	N.D.
PVA:TA 1:0.25	N.D.	N.D.	N.D.	N.D.
PVA:TA 1:0.5	10	20	10	20
PVA:TA 1:1	10	20	5	20

## Data Availability

Data are contained within the article or Supplementary Material.

## Supplementary Materials

The following are available online at https://www.mdpi.com/article/10.3390/polym14010070/s1, Poly(vinyl alcohol)-tannic Acid Cryogel Matrix as Antioxidant and Antibacterial Material. Table S1. The temperatures corresponding to 5% and 10% weight loss of samples.
