# Supplementary material for: Poly(vinyl alcohol)-tannic Acid Cryogel Matrix as Antioxidant and Antibacterial Material
Source: Polymers (Basel). 2021 Dec 25;14(1):70. doi: 10.3390/polym14010070 (PMC8747331; doi:10.3390/polym14010070)
Supplement: Supplementary file 1 [file polymers-14-00070-s001.zip › polymers-1495329-supplementary.pdf]

Poly(vinyl alcohol)-tannic Acid Cryogel Matrix as Antioxidant and Antibacterial Material

Betul Ari[1], Mehtap Sahiner[2], Sahin Demirci[1], Nurettin Sahiner [1,3,4,5]*

1 Department of Chemistry, Faculty of Science & Arts, Canakkale Onsekiz Mart University, Terzioglu Campus, 17100, Canakkale, Turkey.
2 Faculty of Canakkale School of Applied Science, Canakkale Onsekiz Mart University, Terzioglu Campus, Canakkale, 17100, Turkey
3 Nanoscience and Technology Research and Application Center, Canakkale Onsekiz Mart University, Terzioglu Campus, Canakkale 17100, Turkey.
4 Department of Chemical and Biomolecular Engineering, University of South Florida, Tampa, FL 33620, USA.
5 Department of Ophthalmology, Morsani College of Medicine, University of South Florida, 12901 Bruce B Downs B. Downs Blv., MDC 21, Tampa, FL 33612, USA.

*Correspondence: sahiner71@gmail.com (NS)

Hemolysis test

Fresh blood taken from healthy volunteer individuals was used to examine the blood compatibility of cryogel composites. For this, the blood samples taken were placed in hemogram tubes containing EDTA and mildly shaken. From this blood, 2 mL was taken and diluted with 2.5 mL of 0.9% aqueous NaCl solution. Cryogel composite pieces weighing about 10 mg were placed in tubes containing 10 mL of 0.9% aqueous NaCl solution, and 0.2 mL of diluted blood was added slowly to this solution and shaken gently. The blood solution containing cryogel composite was incubated for 1 h in a 37.5 °C oven. For positive and negative control, 0.2 mL of diluted blood was slowly added to tubes containing 10 mL of distilled water and 0.9% aqueous NaCl solution, respectively, and incubated under the same conditions. After these incubated solutions were centrifuged at 100 *g* for 5 min, the absorbance values of the supernatant solution were determined using UV-Vis spectroscopy at 542 nm wavelength. The absorbance value of the solution gives the amount of hemoglobin released as a result of hemolysis of erythrocyte cells. The hemolysis index% of the samples was calculated using Eq. S1.

$$\text{Hemolysis index\%} = [(A_{sample} - A_{negative}) / (A_{positive} - A_{negative})] \times 100 \qquad (S1)$$

where, A_{sample}, $A_{positive}$ and $A_{negative}$ are the absorbance values of sample, positive and negative control, respectively.

Blood coagulation test

Pieces of cryogel composite weighing about 10 mg were placed in a centrifuge tube and swollen with a few drops of 0.9% aqueous NaCl. On this swollen cryogel composite, 0.27 mL was added of the solution prepared by adding 0.064 mL 0.2 M aqueous $CaCl_2$ solution to 0.81 mL fresh blood in another tube. Then the tubes were left to incubate at 37.5 °C for 10 min. After the incubation, 10 mL of pure water kept at 37.5 °C was added slowly onto the blood containing cryogel composite and centrifuged for 1 min at 540 *g*. The non-coagulating part in the tube was diluted by adding into 40 mL of distilled water at 37.5 °C and left for 1 h incubation at 37.5 °C. As a negative control, 0.25 mL of blood was added into 50 mL of

distilled water kept at 37.5 °C and incubated for 1 h under the same conditions. The absorbance values of the incubated solutions were determined by UV-Vis spectroscopy at 542 nm wavelength. The blood coagulation index% of the samples was calculated using Eq. S2.

$$\text{Blood coagulation index} = (A_{sample+\,blood} / A_{blood}) \times 100 \qquad \text{(S2)}$$

where, "$A_{sample+blood}$" is the absorbance value of the blood suspension in contact with the sample, and "A_{blood}" is the absorbance value of the negative control.

All the blood compatibility tests were carried out with three repeats and the results are given as the average values with standard deviations.

where, "$A_{sample+blood}$" is the absorbance value of the blood suspension in contact with the sample, and "A_{blood}" is the absorbance value of the negative control.

All the blood compatibility tests were carried out with three repeat and the results are given as the average values with standard deviations.

Table S1. The temperatures corresponding to 5% and 10% weight loss of samples.

Materials	Weight loss (%)	
	5%	10%
Pure TA	104-203 °C	204-216 °C
Pure Cur	169-233 °C	234-262 °C
PVA cryogel	170-193 °C	194-201 °C
PVA:TA 1:0.1 cryogel/composite	150-193 °C	194-233 °C
PVA:TA 1:0.25 cryogel/composite	132-199 °C	200-252 °C
PVA:TA 1:0.5 cryogel/composite	163-196 °C	197-237 °C
PVA:TA 1:1 cryogel/composite	186-206 °C	207-216 °C
PVA:TA:Cur 1:1:0.1 cryogel/composite	192-210 °C	211-223 °C
